# A convexity‐constrained parameterization of the random effects generalized partial credit model

**DOI:** 10.1111/bmsp.12365

**Published:** 2024-10-27

**Authors:** David J. Hessen

**Affiliations:** ^1^ Department of Methodology and Statistics Utrecht University Utrecht The Netherlands

**Keywords:** expected a posteriori estimates, extended generalized partial credit model, marginal maximum likelihood estimation, random effects generalized partial credit model

## Abstract

An alternative closed‐form expression for the marginal joint probability distribution of item scores under the random effects generalized partial credit model is presented. The closed‐form expression involves a cumulant generating function and is therefore subjected to convexity constraints. As a consequence, complicated moment inequalities are taken into account in maximum likelihood estimation of the parameters of the model, so that the estimation solution is always proper. Another important favorable consequence is that the likelihood function has a single local extreme point, the global maximum. Furthermore, attention is paid to expected a posteriori person parameter estimation, generalizations of the model, and testing the goodness‐of‐fit of the model. Procedures proposed are demonstrated in an illustrative example.

## 1 INTRODUCTION

A well‐known item response model for the measurement of a single latent variable by a set of polytomously scored items with ordered response categories is the generalized partial credit model (Muraki, 1992, 1993). In the generalized partial credit model, the conditional probability distribution of an item score given the latent variable is assumed to depend on the latent variable, an item scaling parameter, and item category location parameters. Well‐known special cases of the generalized partial credit model are the partial credit model (Agresti, 1993; Masters, 1982) and the rating scale model (Andrich, 1978). In the case of dichotomously scored items, the generalized partial credit model equals the two‐parameter logistic model (Birnbaum, 1968) and includes the Rasch model (Rasch, 1960, 1966) as a special case. Generalizations of the generalized partial credit model are the two‐parameter partial credit model (Hemker et al., 1996) and the nominal response model (Bock, 1972).

A distinction can be made between the fixed effects and the random effects generalized partial credit model. In the fixed effects generalized partial credit model, item and person parameters are treated as fixed and can be jointly estimated. Unfortunately, however, joint maximum likelihood estimators of item parameters have been shown to be inconsistent if the number of items is fixed and the number of examinees tends to infinity (Andersen, 1971, 1973; Haberman, 1977). In the random effects generalized partial credit model, the latent variable is assumed to be a random variable with a distribution in the population of examinees and the examinees in the sample are assumed to be randomly sampled from the population. Usually, the latent variable is assumed to have a specific distribution in the population of examinees, and if the generalized partial credit model and the assumed latent variable distribution are true, then item parameters can consistently be estimated in the joint marginal probability distribution of the item scores, where the latent variable has been integrating out.

For the random effects generalized partial credit model without a specified latent variable distribution, it has been shown that the joint marginal probability distribution of the item scores has a closed‐form expression (Hessen, 2021). This closed‐form expression does not seem useful for fitting the random effects generalized partial credit model because of complicated inequality constraints that follow from moment inequalities. However, due to this closed‐form expression, an extended version of the model (Cressie & Holland, 1983; Tjur, 1982) can be fitted to data without requiring the specification of a population distribution for the latent variable and without using numerical integration techniques. An important advantage of using the closed‐form expression in practice is that there will be no estimation bias due to a misspecification of the population distribution of the latent variable. Another advantage is that the model cannot be incorrectly rejected due to a violation of the assumed latent variable distribution. Despite these advantages, the use of this closed‐form expression in fitting the extended generalized partial credit model does not guarantee a solution that is consistent with a latent random variable. Due to ignoring moment inequalities, the estimation solution might not be proper (Cressie & Holland, 1983). Another disadvantage is that in maximum likelihood estimation of the parameters of the extended model, the likelihood function might have several local extreme points.

In this paper, an alternative closed‐form expression of the random effects generalized partial credit model is presented. It is shown that the joint marginal probability distribution of the item scores under the random effects generalized partial model has a closed‐form expression in terms of item category location parameters, item scaling parameters, and an incomplete conditional cumulant‐generating function of the latent random variable given an arbitrary reference score pattern. Since the incomplete cumulant‐generating function is a convex polynomial, the random effects generalized partial credit model can now be fitted to data using this alternative closed‐form expression and appropriate convexity constraints. Fitting the model in this way is latent variable distribution‐free, takes moment inequalities into account, and guarantees a proper estimation solution. Moreover, due to the convexity constraints, the likelihood function now has a single local extreme point, the global maximum.

The framework in which the new closed‐form expression is derived allows the fitting of the random effects generalized partial credit model in a subpopulation defined by a subset of the support of the joint probability distribution of the item scores in the total population (Hessen, 2023). The possibility of fitting a subpopulation model instead of the population model greatly enhances the number of applications in practice because the subpopulation can be chosen so that the normalizing constant in the model expression can efficiently be calculated. For example, if the subpopulation is defined by the set of all observed score patterns, then even when the number of items is large, the number of addends in the denominator of the normalizing constant cannot exceed the sample size. Thus, within this framework, the latent variable distribution‐free application of the random effects generalized partial credit model is not restricted by the number of items.

The remainder of this paper is organized as follows. In the following section, an alternative closed‐form expression of the probability distribution of the item scores under the random effects generalized partial credit model is derived. In the third section, the alternative closed‐form expression is subjected to convexity constraints. In the fourth section, maximum likelihood estimation of the parameters of the random effects generalized partial credit model is discussed. In the fifth section, attention is paid to goodness‐of‐fit testing and to more general models that can be used as alternative models in likelihood ratio tests. One of these more general models is shown to be a convexity‐constrained parameterization of the random effects nominal response model. The sixth section is devoted to expected a posteriori (EAP) estimation of person parameters and to estimating cumulants (e.g., mean, variance, skewness, kurtosis) of the latent variable in the total (sub)population. In the seventh section, an illustrative example is given in which both the distribution of EAP estimates of person parameters and estimates of cumulants in the total (sub)population seem to indicate that the latent variable distribution in the (sub)population is positively skewed and platykurtic.

## 2 A CLOSED‐FORM MODEL EXPRESSION

Consider a situation in which a test of k polytomously scored items is administered to a sample of examinees. The number of response categories of item i is denoted by $m_{i}+1$, where $m_{i}\geq 1$, for all i, and is not restricted to be the same for all i. It is assumed that the examinees are randomly sampled from an infinite population. It is also assumed that the items are measures of a single latent random variable Θ with realization θ. Next, let $Y={\left(Y_{1},\text{…},Y_{k}\right)}^{\prime }$ be the random vector of item scores, and let $y={\left(y_{1},\text{…},y_{k}\right)}^{\prime }$ be a realization, where $y_{i}\in \{0,1,\text{…},m_{i}\}$, for i∈{1,2,…,k}. The joint marginal probability distribution of Y can be written

(1) P(Y=y)=∫P(Y=y|θ)f(θ)dθ,

where P(Y=y|θ) is the joint conditional probability distribution of Y given Θ=θ, and f(θ) is the probability density of Θ in the population of examinees. The elements of Y are assumed to be conditionally independent given Θ=θ. The conditional independence of the elements of Y given Θ=θ is defined as

(2) $$P(Y=y|\theta )=\overset{k}{\underset{i=1}{\prod }}P(Y_{i}=y_{i}|\theta ),$$

where $P(Y_{i}=y_{i}\;|\;\theta )$ is the conditional probability distribution of $Y_{i}$ given Θ=θ. Using dummy scores $x_{is}=1$ if $y_{i}=s$ and $x_{is}=0$ otherwise, for $s\in \{1,\text{…},m_{i}\}$, the conditional probability distribution of $Y_{i}$ given Θ=θ can be written

(3) $$P(Y_{i}=y_{i}|\theta )=P(Y_{i}=0|\theta )\overset{m_{i}}{\underset{s=1}{\prod }}{\left\{V_{is}(\theta )\right\}}^{x_{is}},$$

where $V_{is}(\theta )=P(Y_{i}=s\;|\;\theta )/P(Y_{i}=0\;|\;\theta )$ is the odds of score s on item i relative to score 0 on item i as a function of θ and $P(Y_{i}=0\;|\;\theta )={\left\{1+\overset{m_{i}}{\underset{s=1}{\sum }}V_{is}(\theta )\right\}}^{\;-1}$. The use of the dummy scores in this way fixes the reference category for each item score. The model framework of interest, however, requires flexibility in choosing a different reference category for each item score. By what now follows, this flexibility is obtained. From Eq. 3 it follows for fixed item score $y_{0i}$ that

(4) $$P(Y_{i}=y_{0i}|\theta )=P(Y_{i}=0|\theta )\overset{m_{i}}{\underset{s=1}{\prod }}{\left\{V_{is}(\theta )\right\}}^{x_{0is}},$$

where $x_{0is}=1$ if $y_{0i}=s$ and $x_{0is}=0$ otherwise, for $s=1,\text{…},m_{i}$. Solving for $P(Y_{i}=0\;|\;\theta )$ from Eq. 4 and substitution into Eq. 3 yields

(5) $$P(Y_{i}=y_{i}|\theta )=P(Y_{i}=y_{0i}|\theta )\overset{m_{i}}{\underset{s=1}{\prod }}{\left\{V_{is}(\theta )\right\}}^{{\tilde{x}}_{is}},$$

where ${\tilde{x}}_{is}=x_{is}-x_{0is}$. Next, substitution from Eqs. 2 and 5 into Eq. 1 yields

(6) $$P(Y=y)=\int P(Y=y_{0}|\theta )\left[\overset{k}{\underset{i=1}{\prod }}\overset{m_{i}}{\underset{s=1}{\prod }}{\left\{V_{is}(\theta )\right\}}^{{\tilde{x}}_{is}}\right]f(\theta )\;d\theta ,$$

where $P(Y=y_{0}\;|\;\theta )=\prod_{i=1}^{k}P(Y_{i}=y_{0i}\;|\;\theta )$. Note that

(7) $$V_{is}(\theta )=\overset{s}{\underset{a=1}{\prod }}V_{ia}(\theta ),$$

where $V_{ia}(\theta )=P(Y_{i}=a\;|\;\theta )/P(Y_{i}=a-1\;|\;\theta )$ is the odds of score a on item i relative to score a−1 on item i as a function of θ.

In the generalized partial credit model (Muraki, 1992, 1993) it is assumed that

(8) $$V_{ia}(\theta )=\exp\{\alpha_{i}(\theta -\delta_{ia})\},$$

where $\alpha_{i}$ is an item scaling parameter and $\delta_{ia}$ is an item category location parameter that gives the threshold θ value for which $P(Y_{i}=a-1\;|\;\theta )=P(Y_{i}=a\;|\;\theta )$. Substitution from Eq. 8 into Eq. 7 yields

(9) $$V_{is}(\theta )=\exp\{\alpha_{i}s(\theta -\omega_{is})\},$$

where $\omega_{is}=\sum_{a=1}^{s}\delta_{ia}/s$ is a transformed item category location parameter. The generalized partial credit model specializes to the partial credit model if $\alpha_{i}=\alpha$, for all i (Masters, 1982). In the case of dichotomously scored items, that is $m_{i}=1$, for all i, the generalized partial credit model equals the two‐parameter logistic model (Birnbaum, 1968) and the partial credit model equals the one‐parameter logistic model (Rasch, 1960, 1966).

Now, substitution from Eq. 9 into Eq. 6 gives, after some algebra,

(10) $$P(Y=y)=\exp\left(\overset{k}{\underset{i=1}{\sum }}\overset{m_{i}}{\underset{s=1}{\sum }}\beta_{is}{\tilde{x}}_{is}\right)\int P(Y=y_{0}|\theta )\;\exp\left(\theta \overset{k}{\underset{i=1}{\sum }}\alpha_{i}{\tilde{y}}_{i}\right)f(\theta )\;d\theta ,$$

where $\beta_{is}=-\alpha_{i}s\omega_{is}=-\alpha_{i}\sum_{a=1}^{s}\delta_{ia}$ is another transformed item category location parameter, and ${\tilde{y}}_{i}=y_{i}-y_{0i}$. If it is assumed that f(θ) is the standard normal density, then the integral in Eq. 10 is intractable but can numerically be approximated for any set of parameter values using Gaussian quadrature (Bock & Aitkin, 1981; Bock & Lieberman, 1970). The resulting random effects generalized partial credit model then has $\overset{k}{\underset{i=1}{\sum }}m_{i}+k$ free parameters. Here, however, no specific density is assumed for f(θ). Following Cressie and Holland (1983), we can write

(11) $$P(Y=y)=P(Y=y_{0})\;\exp\left(\overset{k}{\underset{i=1}{\sum }}\beta_{i}^{\prime }{\tilde{x}}_{i}\right)\int \exp\left(\theta \alpha^{\prime }\tilde{y}\right)g(\theta |y_{0})\;d\theta ,$$

where $\beta_{i}={\left(\beta_{i1},\text{…},\beta_{im_{i}}\right)}^{\prime }$, ${\tilde{x}}_{i}={\left({\tilde{x}}_{i1},\text{…},{\tilde{x}}_{im_{i}}\right)}^{\prime }$, $\alpha ={\left(\alpha_{1},\text{…},\alpha_{k}\right)}^{\prime }$, $\tilde{y}={\left({\tilde{y}}_{1},\text{…},{\tilde{y}}_{k}\right)}^{\prime }$, and $g(\theta \;|\;y_{0})=P(Y=y_{0}\;|\;\theta )f(\theta )/P(Y=y_{0})$ is the conditional density of Θ given $Y=y_{0}$. Note that under the partial credit model, $\alpha^{\prime }\tilde{y}=\alpha \tilde{y}$, where $\tilde{y}=\sum_{i=1}^{k}{\tilde{y}}_{i}$. Subsequently, Eq. 11 can alternatively be written

(12) $$P(Y=y)=P(Y=y_{0})\;\exp\left(\beta^{\prime }\tilde{x}\right)M_{\Theta |y_{0}}(\alpha^{\prime }\tilde{y}),$$

where $\beta =\text{vec}(\beta_{1},\text{…},\beta_{k})$, $\tilde{x}=\text{vec}({\tilde{x}}_{1},\text{…},{\tilde{x}}_{k})$, and $M_{\Theta |y_{0}}(\alpha^{\prime }\tilde{y})=E\{\exp(\alpha^{\prime }\tilde{y}\Theta )\;|\;y_{0}\}$ is the conditional moment‐generating function of Θ given $Y=y_{0}$. It is well known that

(13) $$M_{\Theta |y_{0}}(\alpha^{\prime }\tilde{y})=\exp\{K_{\Theta |y_{0}}(\alpha^{\prime }\tilde{y})\}=\exp\left\{\overset{\infty }{\underset{r=1}{\sum }}\kappa_{r}\frac{1}{r!}{\left(\alpha^{\prime }\tilde{y}\right)}^{r}\right\},$$

where $K_{\Theta |y_{0}}(\alpha^{\prime }\tilde{y})$ is the conditional cumulant‐generating function of Θ given $Y=y_{0}$, and $\kappa_{r}$ is the rth cumulant of Θ given $Y=y_{0}$. The first cumulant $\kappa_{1}$ is the conditional mean of Θ given $Y=y_{0}$, the second cumulant $\kappa_{2}$ is the conditional variance of Θ given $Y=y_{0}$, and the third cumulant $\kappa_{3}$ is the third central moment of Θ given $Y=y_{0}$. Note that $\kappa_{3}$ determines the skewness of the conditional distribution of Θ given $Y=y_{0}$ because if $\kappa_{3}<0$, then the coefficient of skewness given by $\kappa_{3}/\kappa_{2}^{3/2}$ is negative and the distribution is skewed to the left, and if $\kappa_{3}>0$, the coefficient of skewness is positive and the distribution is skewed to the right. Also note that $\kappa_{4}$ determines the kurtosis of the distribution of Θ given $Y=y_{0}$ because if $\kappa_{4}<0$, then the coefficient of kurtosis given by $3+\kappa_{4}/\kappa_{2}^{2}$ is less than 3 and the distribution is platykurtic; if $\kappa_{4}=0$, then the coefficient of kurtosis is 3 and the distribution is mesokurtic; and if $\kappa_{4}>0$, then the coefficient of kurtosis is greater than 3 and the distribution is leptokurtic.

Substitution from Eq. 13 into Eq. 11 yields an unidentifiable model with infinitely many parameters. Hessen (2021) has shown, however, that the conditional moment‐generating function of Θ given Y=0 has a closed‐form expression in terms of common assocation parameters. Although this closed‐form expression yields an identifiable model after fixing the scale of θ, the expression is quite complex and its parameters are not very informative about the conditional distribution of Θ given Y=0. The result in the following theorem provides a closed‐form expression for the conditional moment‐generating function of Θ given $Y=y_{0}$. This closed‐form expression for $M_{\Theta |y_{0}}(\alpha^{\prime }\tilde{y})$ is simpler and better interpretable than the closed‐form expression for $M_{\Theta |0}(\alpha^{\prime }y)$ proposed by Hessen (2021) and also yields an identifiable model.

Theorem 1 Under the model in Eq. 12, the conditional moment‐generating function of Θ given $Y=y_{0}$ equals

(14) $$M_{\Theta |y_{0}}(\alpha^{\prime }\tilde{y})=\exp\left\{\overset{v}{\underset{r=1}{\sum }}\lambda_{r}\frac{1}{r!}{\left(\alpha^{\prime }\tilde{y}\right)}^{r}\right\},$$

where $\lambda_{1},\text{…},\lambda_{v-1}$ are the first v−1 conditional cumulants of Θ given $Y=y_{0}$, and $\lambda_{v}=K_{\Theta |y_{0}}^{\left(v\right)}(\xi_{v})$ is the vth derivative of $K_{\Theta |y_{0}}(\alpha^{\prime }\tilde{y})=\ln M_{\Theta |y_{0}}(\alpha^{\prime }\tilde{y})$ for some real number $\xi_{v}$ between zero and $\alpha^{\prime }\tilde{y}$.

The (v−1)th‐order Maclaurin polynomial of $K_{\Theta |y_{0}}(\alpha^{\prime }\tilde{y})$ is

(15) $$\overset{v-1}{\underset{r=0}{\sum }}K_{\Theta |y_{0}}^{\left(r\right)}(0)\frac{1}{r!}{\left(\alpha^{\prime }\tilde{y}\right)}^{r}=\overset{v-1}{\underset{r=1}{\sum }}\kappa_{r}\frac{1}{r!}{\left(\alpha^{\prime }\tilde{y}\right)}^{r},$$

where $K_{\Theta |y_{0}}^{\left(r\right)}(0)$ is the rth derivative of $K_{\Theta |y_{0}}(\alpha^{\prime }\tilde{y})$ at $\alpha^{\prime }\tilde{y}=0$. The Lagrange form of the remainder is given by

(16) $$R_{v-1}(\alpha^{\prime }\tilde{y})=K_{\Theta |y_{0}}(\alpha^{\prime }\tilde{y})-\overset{v-1}{\underset{r=1}{\sum }}\kappa_{r}\frac{1}{r!}{\left(\alpha^{\prime }\tilde{y}\right)}^{r}=K_{\Theta |y_{0}}^{\left(v\right)}(\xi_{v})\frac{1}{v!}{\left(\alpha^{\prime }\tilde{y}\right)}^{v}$$

for some real number $\xi_{v}$ between zero and $\alpha^{\prime }\tilde{y}$. Solving Eq. 16 for $K_{\Theta |y_{0}}(\alpha^{\prime }\tilde{y})$ and substitution into Eq. 13 yields Eq. 14, where $\lambda_{r}=\kappa_{r}$, for r=1,…,v−1, and $\lambda_{v}=K_{\Theta |y_{0}}^{\left(v\right)}(\xi_{v})$. This completes the proof. □

Note that v is a positive integer that must be specified. If v>2, then the moment‐generating function in Eq. 14 is incomplete and can only generate the first v−1 cumulants. In the case v=1 or $\lambda_{r}=0$, for r≥2, the distribution of Θ given $Y=y_{0}$ is degenerate and the model equals the independence model. The special case in the following theorem is a reparameterization of the conditional normal generalized partial credit model (Hessen, 2021). In this special case, v=2 and $\lambda_{2}>0$.

Theorem 2 If all conditional cumulants given $Y=y_{0}$ of order r>2 are zero, then the conditional distribution of Θ given Y=y is normal with mean $\kappa_{1}+\kappa_{2}\alpha^{\prime }\tilde{y}$ (linear) and variance $\kappa_{2}$ (homoscedastic).

If $\kappa_{r}=0$, for r>2, then

$$M_{\Theta |y_{0}}(\alpha^{\prime }\tilde{y})=\exp\left\{\kappa_{1}\alpha^{\prime }\tilde{y}+\kappa_{2}\frac{1}{2}{\left(\alpha^{\prime }\tilde{y}\right)}^{2}\right\},$$

which is the moment‐generating function of a normal random variable with mean $\kappa_{1}$ and variance $\kappa_{2}$. Furthermore,

(17) $$g(\theta |y)=\frac{P(Y=y|\theta )f(\theta )}{P(Y=y)}=\frac{\exp(\theta \alpha^{\prime }\tilde{y})P(Y=y_{0}|\theta )f(\theta )}{M_{\Theta |y_{0}}(\alpha^{\prime }\tilde{y})P(Y=y_{0})}=\frac{\exp(\theta \alpha^{\prime }\tilde{y})}{M_{\Theta |y_{0}}(\alpha^{\prime }\tilde{y})}g(\theta |y_{0}),$$

where $g(\theta \;|\;y_{0})={\left(2\pi \kappa_{2}\right)}^{-1/2}\exp\{-\frac{1}{2}{\left(\theta -\kappa_{1}\right)}^{2}/\kappa_{2}\}$, so that after some algebra it follows that

$$g(\theta |y)={\left(2\pi \kappa_{2}\right)}^{-1/2}\exp[-\frac{1}{2}{\left\{\theta -(\kappa_{1}+\kappa_{2}\alpha^{\prime }\tilde{y})\right\}}^{2}/\kappa_{2}],$$

which can be recognized as a normal density with mean $\kappa_{1}+\kappa_{2}\alpha^{\prime }\tilde{y}$ and variance $\kappa_{2}$. □

Substitution from Eq. 14 into Eq. 12 gives

(18) $$P(Y=y)=\tau \;\exp\left\{\beta^{\prime }\tilde{x}+\overset{v}{\underset{r=1}{\sum }}\lambda_{r}\frac{1}{r!}{\left(\alpha^{\prime }\tilde{y}\right)}^{r}\right\},$$

where $\tau =P(Y=y_{0})$ is a normalizing constant. This normalizing constant equals

(19) $$\tau ={\left[\underset{y\in S}{\sum }\exp\left\{\beta^{\prime }\tilde{x}+\overset{v}{\underset{r=1}{\sum }}\lambda_{r}\frac{1}{r!}{\left(\alpha^{\prime }\tilde{y}\right)}^{r}\right\}\right]}^{-1},$$

where S={y|P(Y=y)>0} is the support of Y, that is, the set of all score patterns that are observable. Without additional constraints the model is not identifiable. In the case v≥2, there are two indeterminacies. To show these two indeterminacies, the exponent on the right‐hand side of Eq. 18 is rewritten as $\overset{k}{\underset{i=1}{\sum }}\overset{m_{i}}{\underset{s=1}{\sum }}(\beta_{is}+\lambda_{1}\alpha_{i}s){\tilde{x}}_{is}+\overset{v}{\underset{r=2}{\sum }}\lambda_{r}\frac{1}{r!}{\left(\alpha^{\prime }\tilde{y}\right)}^{r}$. It follows that if $\beta_{is}=\beta_{is}^{*}+c\alpha_{i}s$ and $\lambda_{1}=\lambda_{1}^{*}-c$, for some constant c, then $\beta_{is}+\lambda_{1}\alpha_{i}s=\beta_{is}^{*}+\lambda_{1}^{*}\alpha_{i}s$. In addition, if $\alpha =\alpha^{*}c^{-1}$ and $\lambda_{r}=\lambda_{r}^{*}c^{r}$, for some constant c, then ${\left({\tilde{y}}^{\prime }\alpha \right)}^{r}\lambda_{r}={\left({\tilde{y}}^{\prime }\alpha^{*}\right)}^{r}\lambda_{r}^{*}$. The two indeterminacies can be solved by arbitrarily setting $\lambda_{1}=0$ and $\lambda_{2}=1$. Consequently, the number of free parameters is $\overset{k}{\underset{i=1}{\sum }}m_{i}+k+v-2$, for v≥2, and since $\lambda_{v}$ is not a cumulant, only v−3 of the free parameters are conditional cumulants, for v≥3. Note that sign indeterminacy remains because ${\left({\tilde{y}}^{\prime }\alpha \right)}^{r}\lambda_{r}={\left({\tilde{y}}^{\prime }\alpha \right)}^{r}{\left(-1\right)}^{r}\cdot -\lambda_{r}$, for odd r. In the random effects partial credit model, the right‐hand side of Eq. 18 equals $\overset{k}{\underset{i=1}{\sum }}\overset{m_{i}}{\underset{s=1}{\sum }}\phi_{is}{\tilde{x}}_{is}+\overset{v}{\underset{r=2}{\sum }}\psi_{r}\frac{1}{r!}{\tilde{y}}^{r}$, where $\phi_{is}=\beta_{is}+\lambda_{1}\alpha s$, for all i and s, and $\psi_{r}=\lambda_{r}\alpha^{r}$, for r=2,…,v, are identifiable. In this model, the number of free parameters is $\overset{k}{\underset{i=1}{\sum }}m_{i}+v-1$.

## 3 CONVEXITY CONSTRAINTS

It follows from Hölder's inequality that the cumulant‐generating function $K_{\Theta |y_{0}}(\alpha^{\prime }\tilde{y})=\sum_{r=1}^{v}\lambda_{r}\frac{1}{r!}{\left(\alpha^{\prime }\tilde{y}\right)}^{r}$ is a convex polynomial. This polynomial is of degree v and has v coefficients. Consequently, its first derivative

(20) $$K_{\Theta |y_{0}}^{\left(1\right)}(\alpha^{\prime }\tilde{y})=\overset{v}{\underset{r=1}{\sum }}\lambda_{r}\frac{1}{(r-1)!}{\left(\alpha^{\prime }\tilde{y}\right)}^{r-1}$$

is a strictly increasing polynomial of degree v−1, also with v coefficients, and its second derivative

(21) $$K_{\Theta |y_{0}}^{\left(2\right)}(\alpha^{\prime }\tilde{y})=\overset{v}{\underset{r=2}{\sum }}\lambda_{r}\frac{1}{(r-2)!}{\left(\alpha^{\prime }\tilde{y}\right)}^{r-2}$$

is a non‐negative polynomial of degree v−2, with v−1 coefficients. It is well known that $K_{\Theta |y_{0}}^{\left(2\right)}(\alpha^{\prime }\tilde{y})$ is non‐negative on (−∞,∞) if and only if v−2=2q, where v is even, and

(22) $$K_{\Theta |y_{0}}^{\left(2\right)}(\alpha^{\prime }\tilde{y})=p_{1}{\left(\alpha^{\prime }\tilde{y}\right)}^{2}+p_{2}{\left(\alpha^{\prime }\tilde{y}\right)}^{2},$$

where $p_{1}(\alpha^{\prime }\tilde{y})$ and $p_{2}(\alpha^{\prime }\tilde{y})$ are polynomials whose degrees are at most q∈{0,1,2,…} (Brickman & Steinberg, 1962; Murray et al., 2016). If both polynomials are of degree q and each has q+1 coefficients, then the 2q+1 coefficients of the resulting polynomial of degree 2q in Eq. 22 are functions of 2q+2 parameters. Here, $p_{1}(\alpha^{\prime }\tilde{y})$ and $p_{2}(\alpha^{\prime }\tilde{y})$ should be chosen so that the coefficients of the resulting polynomial are functions of at most v−2=2q free parameters. Thus, at least two constraints are needed for identification.

Let $p_{1}(\alpha^{\prime }\tilde{y})=\epsilon_{1}^{\prime }t$ and $p_{2}(\alpha^{\prime }\tilde{y})=\epsilon_{2}^{\prime }t$, where $\epsilon_{1}={\left(\epsilon_{10},\epsilon_{11},\text{…},\epsilon_{1q}\right)}^{\prime }$, $\epsilon_{2}={\left(\epsilon_{20},\epsilon_{21},\text{…},\epsilon_{2q}\right)}^{\prime }$, and $t={\left(1,\alpha^{\prime }\tilde{y},{\left(\alpha^{\prime }\tilde{y}\right)}^{2},\text{…},{\left(\alpha^{\prime }\tilde{y}\right)}^{q}\right)}^{\prime }$. Then, $K_{\Theta |y_{0}}^{\left(2\right)}(\alpha^{\prime }\tilde{y})={\left(\epsilon_{1}^{\prime }t\right)}^{2}+{\left(\epsilon_{2}^{\prime }t\right)}^{2}$ equals

(23) $$\begin{array}{ll} K_{\Theta |y_{0}}^{\left(2\right)}(\alpha^{\prime }\tilde{y}) & =t^{\prime }(\epsilon_{1}\epsilon_{1}^{\prime }+\epsilon_{2}\epsilon_{2}^{\prime })t\; \end{array}$$

(24) $$\begin{array}{ll} & =\overset{q}{\underset{j=0}{\sum }}\overset{q}{\underset{j^{\prime }=0}{\sum }}(\epsilon_{1j}\epsilon_{1j^{\prime }}+\epsilon_{2j}\epsilon_{2j^{\prime }}){\left(\alpha^{\prime }\tilde{y}\right)}^{j+j^{\prime }}\; \end{array}$$

(25) $$\begin{array}{ll} & =\overset{2q}{\underset{c=0}{\sum }}h_{c}(\epsilon_{1},\epsilon_{2}){\left(\alpha^{\prime }\tilde{y}\right)}^{c},\; \end{array}$$

where $h_{c}(\epsilon_{1},\epsilon_{2})=\underset{j+j^{\prime }=c}{\sum }(\epsilon_{1j}\epsilon_{1j^{\prime }}+\epsilon_{2j}\epsilon_{2j^{\prime }})=\frac{1}{c!}\lambda_{c+2}=\frac{1}{(r-2)!}\lambda_{r}$. As a consequence, we can write

(26) $$\begin{array}{ll} K_{\Theta |y_{0}}^{\left(1\right)}(\alpha^{\prime }\tilde{y}) & =\lambda_{1}+\int_{0}^{\alpha^{\prime }\tilde{y}}\overset{2q}{\underset{c=0}{\sum }}h_{c}(\epsilon_{1},\epsilon_{2})\;w^{c}\;dw\; \end{array}$$

(27) $$\begin{array}{ll} & =\lambda_{1}+\overset{2q}{\underset{c=0}{\sum }}h_{c}(\epsilon_{1},\epsilon_{2})\int_{0}^{\alpha^{\prime }\tilde{y}}w^{c}\;dw\; \end{array}$$

(28) $$\begin{array}{ll} & =\lambda_{1}+\overset{2q}{\underset{c=0}{\sum }}h_{c}(\epsilon_{1},\epsilon_{2})\frac{1}{c+1}{\left(\alpha^{\prime }\tilde{y}\right)}^{c+1},\; \end{array}$$

and the cumulant‐generating function can be written

(29) $$\begin{array}{ll} K_{\Theta |y_{0}}(\alpha^{\prime }\tilde{y}) & =\lambda_{1}\alpha^{\prime }\tilde{y}+\overset{2q}{\underset{c=0}{\sum }}h_{c}(\epsilon_{1},\epsilon_{2})\frac{1}{c+1}\int_{0}^{\alpha^{\prime }\tilde{y}}w^{c+1}\;dw\\ & =\lambda_{1}\alpha^{\prime }\tilde{y}+\overset{2q}{\underset{c=0}{\sum }}h_{c}(\epsilon_{1},\epsilon_{2})\frac{1}{c+1}\frac{1}{c+2}{\left(\alpha^{\prime }\tilde{y}\right)}^{c+2},\; \end{array}$$

where $\lambda_{1}$ is set to zero for identification. If, in addition, $\left(\epsilon_{10},\epsilon_{20}\right)$ is arbitrarily set to $\left(\frac{1}{\sqrt{2}},\frac{1}{\sqrt{2}}\right)$ or $\left(\frac{3}{5},\frac{4}{5}\right)$, then $h_{0}(\epsilon_{1},\epsilon_{2})=\epsilon_{10}^{2}+\epsilon_{20}^{2}=\lambda_{2}=1$, and the model is identified. More constraints can be added, however, while keeping the polynomial in Eq. 22 of degree 2q. If more constraints are added, then $\lambda_{3},\text{…},\lambda_{v}$ are modeled by a smaller number of parameters than v−2. For example, if the degree of $p_{1}(\alpha^{\prime }\tilde{y})$ is q and the degree of $p_{2}(\alpha^{\prime }\tilde{y})$ is q−1, then the degree of the resulting polynomial in Eq. 22 is still 2q, but its 2q+1 coefficients are now functions of 2q+1 parameters, of which only 2q−1 are free. Note that the elements of the parameter vectors $\epsilon_{1}$ and $\epsilon_{2}$ do not have a statistically meaningful interpretation. These elements should be conceived of as auxiliary parameters that are used only to constrain $\lambda_{2},\text{…},\lambda_{v}$ such that the conditional cumulant‐generating function of Θ given $Y=y_{0}$ is convex.

## 4 MAXIMUM LIKELIHOOD ESTIMATION

The random effects generalized partial credit model given by

(30) $$P(Y=y)=\tau \;\exp\left\{\beta^{\prime }\tilde{x}+K_{\Theta |y_{0}}(\alpha^{\prime }\tilde{y})\right\},$$

where $K_{\Theta |y_{0}}(\alpha^{\prime }\tilde{y})$ is given by Eq. 29, is the population model for which the normalizing constant equals

(31) $$\tau ={\left[\underset{y\in S}{\sum }\exp\left\{\beta^{\prime }\tilde{x}+K_{\Theta |y_{0}}(\alpha^{\prime }\tilde{y})\right\}\right]}^{-1},$$

where S is the support of Y. S might be an unknown proper subset of the set of all theoretically possible score patterns. Note that it follows from Eq. 12 that $y_{0}$ must be an element of S; otherwise, the model is degenerate. Let O⊆B⊆S, where O is the set of all observed score patterns in the sample. Thus, selecting $y_{0}\in O$ guarantees that $y_{0}\in S$. According to Hessen (2023), it follows from the population model in Eq. 30 that

(32) $$P(Y=y|B)=\tau_{B}\;\exp\left\{\beta^{\prime }\tilde{x}+K_{\Theta |y_{0}}(\alpha^{\prime }\tilde{y})\right\},$$

where

(33) $$\tau_{B}={\left[\underset{y\in B}{\sum }\exp\left\{\beta^{\prime }\tilde{x}+K_{\Theta |y_{0}}(\alpha^{\prime }\tilde{y})\right\}\right]}^{-1}.$$

Now, let $n_{y}$ be the number of examinees in the sample with score pattern y, for all y. Then, taking all possible score patterns into account and assuming the independence of observations, the likelihood function for the subpopulation model in Eq. 32 is given by

(34) $$L=\tau_{B}^{n}\;\exp\left\{\beta^{\prime }\tilde{n}+\underset{y\in O}{\sum }n_{y}K_{\Theta |y_{0}}(\alpha^{\prime }\tilde{y})\right\},$$

where n is the total sample size, and $\tilde{n}=\underset{y}{\sum }n_{y}\tilde{x}$. For fixed α and without convexity constraints, the likelihood function for the model in Eq. 18 is log‐concave in the remaining parameters because for fixed α, the model is a member of the exponential family. Now, since $K_{\Theta |y_{0}}(\alpha^{\prime }\tilde{y})$ is convex in $\alpha^{\prime }\tilde{y}$ and therefore also convex in α, the likelihood function in Eq. 34 is log‐concave in all parameters. This means that the likelihood function has a single local extreme point, the global maximum. To find the estimates of the elements of β, α, $\epsilon_{1}$, and $\epsilon_{2}$ that maximize the likelihood function in Eq. 34, the log‐likelihood function can be maximized with respect to the parameters subject to identification constraints using the Broyden‐Fletcher‐Goldfarb‐Shanno (BFGS) algorithm (Fletcher, 1987). The BFGS algorithm is a quasi‐Newton method in which the Hessian matrix of second derivatives is approximated using updates specified by (approximate) evaluations of the first derivatives. The first derivatives of the log‐likelihood function with respect to β, α, and the elements of $\epsilon_{1}$ and $\epsilon_{2}$ are given in Appendix.

Let ${\hat{\beta }}_{i}$ and ${\hat{\alpha }}_{i}$ be the maximum likelihood estimates of $\beta_{i}$ and $\alpha_{i}$, respectively, for all i. Then the maximum likelihood estimate of the threshold vector $\delta_{i}={\left(\delta_{i1},\text{…},\delta_{im_{i}}\right)}^{\prime }$ is ${\hat{\delta }}_{i}=-{\hat{\alpha }}_{i}^{-1}J_{i}^{-1}{\hat{\beta }}_{i}$, for all i, where $J_{i}$ is a lower triangular matrix whose diagonal entries and entries below the main diagonal are all one, for all i. Let ${\hat{\epsilon }}_{1}$ and ${\hat{\epsilon }}_{2}$ be the maximum likelihood estimates of $\epsilon_{1}$ and $\epsilon_{2}$, respectively. Then the maximum likelihood estimate of $\lambda_{r}$ is ${\hat{\lambda }}_{r}=h_{r-2}({\hat{\epsilon }}_{1},{\hat{\epsilon }}_{2})(r-2)!$, for r∈{2,…,v}.

## 5 PERSON AND POPULATION PARAMETERS

A popular way of obtaining person parameter estimates under a random effects item response model is the calculation of the so‐called EAP estimates. An EAP estimate is an estimate of the conditional latent mean given score pattern y. The calculation of such an estimate requires a theoretical expression for the conditional latent mean given score pattern y. The theoretical expression of this conditional latent mean can be obtained via the moment‐generating function of the latent variable given score pattern y. This conditional moment‐generating function is given in the following theorem.

Theorem 3 Under the random effects generalized partial credit response model, the conditional moment‐generating function of Θ given Y=y is

(35) $$M_{\Theta |y}(z)=\exp\left[\overset{v}{\underset{r=1}{\sum }}\lambda_{r}\frac{1}{r!}\left\{{\left(z+\alpha^{\prime }\tilde{y}\right)}^{r}-{\left(\alpha^{\prime }\tilde{y}\right)}^{r}\right\}\right].$$

The conditional moment‐generating function of Θ given Y=y is given by

(36) $$M_{\Theta |y}(z)=\int \exp(z\theta )g(\theta |y)\;d\theta .$$

Substitution from Eq. 17 into Eq. 36 yields

$$\begin{array}{ll} M_{\Theta |y}(z) & =\int \exp(z\theta )\exp(\alpha^{\prime }\tilde{y}\theta )g(\theta |y_{0})\;d\theta \;{\left\{M_{\Theta |y_{0}}(\alpha^{\prime }\tilde{y})\right\}}^{-1}\;\\ & =\int \exp\{(z+\alpha^{\prime }\tilde{y})\theta \}g(\theta |y_{0})\;d\theta \;{\left\{M_{\Theta |y_{0}}(\alpha^{\prime }\tilde{y})\right\}}^{-1}\;\\ & =M_{\Theta |y_{0}}(z+\alpha^{\prime }\tilde{y}){\left\{M_{\Theta |y_{0}}(\alpha^{\prime }\tilde{y})\right\}}^{-1}\;\\ & =\exp\left\{\overset{v}{\underset{r=1}{\sum }}\lambda_{r}\frac{1}{r!}{\left(z+\alpha^{\prime }\tilde{y}\right)}^{r}-\overset{v}{\underset{r=1}{\sum }}\lambda_{r}\frac{1}{r!}{\left(\alpha^{\prime }\tilde{y}\right)}^{r}\right\},\; \end{array}$$

and factorizing $\overset{v}{\underset{r=1}{\sum }}{\left(r!\right)}^{-1}$ gives Eq. 35. □

The jth derivative of $K_{\Theta |y}(z)=\ln M_{\Theta |y}(z)$ with respect to z can be shown to be equal to

(37) $$K_{\Theta |y}^{\left(j\right)}(z)=\overset{v}{\underset{r=j}{\sum }}\lambda_{r}\frac{1}{(r-j)!}{\left(z+\alpha^{\prime }\tilde{y}\right)}^{r-j}.$$

Consequently, the jth conditional cumulant of Θ given Y=y is given by

(38) $$K_{\Theta |y}^{\left(j\right)}(0)=\kappa_{jy}=\overset{v}{\underset{r=j}{\sum }}\lambda_{r}\frac{1}{(r-j)!}{\left(\alpha^{\prime }\tilde{y}\right)}^{r-j}.$$

$K_{\Theta |y}^{\left(1\right)}(0)=\mu_{y}$ and $K_{\Theta |y}^{\left(2\right)}(0)=\sigma_{y}^{2}$ are the conditional mean and variance of Θ given Y=y, respectively. Note that since $\mu_{y}$ is exactly equal to $K_{\Theta |y_{0}}^{\left(1\right)}(\alpha^{\prime }\tilde{y})$ in Eq. 20, it is a strictly increasing function of $\alpha^{\prime }\tilde{y}$. Also note that since $\sigma_{y}^{2}$ is exactly equal to $K_{\Theta |y_{0}}^{\left(2\right)}(\alpha^{\prime }\tilde{y})$ in Eq. 21, it is a non‐negative function of $\alpha^{\prime }\tilde{y}$. The maximum likelihood estimate of the jth conditional cumulant of Θ given Y=y is given by

$${\hat{\kappa }}_{jy}=\overset{v}{\underset{r=j}{\sum }}{\hat{\lambda }}_{r}\frac{1}{(r-j)!}{\left({\hat{\alpha }}^{\prime }\tilde{y}\right)}^{r-j},\;\;\text{for all}\;y,$$

where ${\hat{\lambda }}_{r}$ is as before and $\hat{\alpha }$ is the maximum likelihood estimate of α. The maximum likelihood estimate of $\mu_{y}$ is the EAP person parameter estimate given by ${\hat{\kappa }}_{1y}={\hat{\mu }}_{y}$, for all y. The maximum likelihood estimate of $\sigma_{y}^{2}$ is ${\hat{\kappa }}_{2y}={\hat{\sigma }}_{y}^{2}$, for all y.

It is well known that the jth conditional non‐central moment $E(\Theta^{j}\;|\;y)=\mu_{jy}^{\prime }$ is a jth‐degree polynomial in the first j conditional cumulants of Θ given Y=y. The first four conditional non‐central moments as polynomial functions of the first four conditional cumulants are given in Appendix. By the law of total expectation, it then follows that the jth non‐central moment of Θ in the total population defined by B is given by

(39) $$E(\Theta^{j}|B)=\mu_{jB}^{\prime }=\underset{y\in B}{\sum }\mu_{jy}^{\prime }P(Y=y|B),\;\;\text{for}\;j\in \{1,\text{…},v-1\}.$$

Then the first four cumulants of Θ (mean, variance, skewness, and kurtosis) in the population defined by B are given by

$$\begin{array}{ll} \kappa_{1B} & =\mu_{1B}^{\prime },\;\\ \kappa_{2B} & =\mu_{2B}^{\prime }-{\left(\mu_{1B}^{\prime }\right)}^{2},\;\\ \kappa_{3B} & =\mu_{3B}^{\prime }-3\mu_{2B}^{\prime }\mu_{1B}^{\prime }+2{\left(\mu_{1B}^{\prime }\right)}^{3},\;\\ \kappa_{4B} & =\mu_{4B}^{\prime }-4\mu_{3B}^{\prime }\mu_{1B}^{\prime }-3{\left(\mu_{2B}^{\prime }\right)}^{3}+12\mu_{2B}^{\prime }{\left(\mu_{1B}^{\prime }\right)}^{2}-6{\left(\mu_{1B}^{\prime }\right)}^{4},\; \end{array}$$

respectively. Thus, with the maximum likelihood estimates of the first four conditional cumulants of Θ given Y=y, for all y∈B, maximum likelihood estimates of the first four conditional non‐central moments of Θ given Y=y can be calculated, for all y∈B. Subsequently, the maximum likelihood estimates of the first four non‐central moments of Θ in the population defined by B can be calculated using Eq. 39 and, finally, the maximum likelihood estimates of the first four cumulants of Θ in the population defined by B.

## 6 GOODNESS OF FIT AND GENERALIZATIONS

For assessing the goodness of fit of a random effects generalized partial credit model in the subpopulation defined by B⊇O, Pearson's asymptotic chi‐squared test or the likelihood ratio test against the saturated model might be appropriate. However, these tests often cannot be validly applied due to too many observed score patterns with low sample frequencies. Better goodness‐of‐fit tests in these circumstances are the alternative likelihood ratio tests proposed by Hessen (2023).

In addition, a random effects generalized partial credit model can be tested against less general alternatives than the saturated model using likelihood ratio tests. One such less general alternative is the model given by

(40) $$P(Y=y|B)=\tau_{B}\;\exp\left\{\overset{k}{\underset{i=1}{\sum }}\overset{m_{i}}{\underset{s=1}{\sum }}\nu_{is}{\tilde{x}}_{is}+\underset{i<i^{\prime }}{\sum }\rho_{ii^{\prime }}{\tilde{y}}_{i}{\tilde{y}}_{i^{\prime }}+\overset{v}{\underset{r=3}{\sum }}\lambda_{r}\frac{1}{r!}{\left(\alpha^{\prime }\tilde{y}\right)}^{r}\right\}.$$

This model specializes to the random effects generalized partial credit model if $\nu_{is}=\beta_{is}+\lambda_{1}\alpha_{i}s+\frac{1}{2}\lambda_{2}\alpha_{i}^{2}s^{2}$, $\rho_{ij}=\lambda_{2}\alpha_{i}\alpha_{j}$, and $\lambda_{r}=h_{r-2}(\epsilon_{1},\epsilon_{2})(r-2)!$, for r≥2. Another less general alternative is the random effects nominal response model (Bock, 1972) given by

(41) $$P(Y=y|B)=\tau_{B}\;\exp\left\{\beta^{\prime }\tilde{x}+\overset{2q}{\underset{c=0}{\sum }}h_{c}(\epsilon_{1},\epsilon_{2})\frac{1}{c+1}\frac{1}{c+2}{\left(\eta^{\prime }\tilde{x}\right)}^{c+2}\right\},$$

where η is a vector of unrestricted item category scaling parameters. Note that if $\eta^{\prime }\tilde{x}=\overset{k}{\underset{i=1}{\sum }}\overset{m_{i}}{\underset{s=1}{\sum }}\eta_{is}x_{is}$, where $\eta_{is}=\alpha_{i}s$, for all i and s, then this model specializes to the random effects generalized partial credit model. A third less general alternative is the model given by

(42) $$P(Y=y|B)=\tau_{B}\;\exp\left\{\overset{k}{\underset{i=1}{\sum }}\overset{m_{i}}{\underset{s=1}{\sum }}\nu_{is}{\tilde{x}}_{is}+\underset{i<i^{\prime }}{\sum }\overset{m_{i}}{\underset{s=1}{\sum }}\overset{m_{i^{\prime }}}{\underset{s^{\prime }=1}{\sum }}\rho_{isi^{\prime }s^{\prime }}{\tilde{x}}_{is}{\tilde{x}}_{i^{\prime }s^{\prime }}+\overset{v}{\underset{r=3}{\sum }}\lambda_{r}\frac{{\left(\eta^{\prime }\tilde{x}\right)}^{r}}{r!}\right\}.$$

This model specializes to the random effects nominal response model in Eq. 41 if $\nu_{is}=\beta_{is}+\lambda_{1}\eta_{is}+\frac{1}{2}\lambda_{2}\eta_{is}^{2}$, $\rho_{isi^{\prime }s^{\prime }}=\lambda_{2}\eta_{is}\eta_{i^{\prime }s^{\prime }}$, for all $i,s,i^{\prime },s^{\prime }$, and $\lambda_{r}=h_{r-2}(\epsilon_{1},\epsilon_{2})(r-2)!$, for r≥2. Note that the model in Eq. 42 can also be used as an alternative in a likelihood ratio test of the random effects nominal response model in Eq. 41.

## 7 AN ILLUSTRATIVE EXAMPLE

The data in this example are the responses of 493 adolescents to five polytomously scored items that are intended to measure aggressive antisocial behaviour (Dekovic, 2003). The adolescents were asked to indicate on a five‐point scale how often they have committed the antisocial act given by each item in the last 12 months. The responses are coded as 0 = *never*, 1 = *once*, 2 = *two or three times*, 3 = *four to ten times*, and 4 = *more than ten times*. Thus, $m_{i}=4$, for all i. All observed score patterns and their observed frequencies are given in Table 1.

**TABLE 1:** Aggressive antisocial behaviour data (Dekovic, 2003): score patterns, observed frequencies, EAPs, and conditional latent variances.

y	$n_{y}$	${\hat{\mu }}_{y}$	${\hat{\sigma }}_{y}^{2}$	y	$n_{y}$	${\hat{\mu }}_{y}$	${\hat{\sigma }}_{y}^{2}$	y	$n_{y}$	${\hat{\mu }}_{y}$	${\hat{\sigma }}_{y}^{2}$
${\left(0,\;0,\;0,\;0,\;0\right)}_{1}$	302	0.000	1.000	${\left(0,\;2,\;0,\;2,\;0\right)}_{2}$	1	2.711	0.199	${\left(0,\;4,\;0,\;4,\;1\right)}_{1}$	1	3.068	0.005
${\left(1,\;0,\;0,\;0,\;0\right)}_{2}$	7	1.172	0.692	${\left(1,\;2,\;0,\;2,\;0\right)}_{2}$	1	2.919	0.105	${\left(0,\;4,\;2,\;4,\;1\right)}_{2}$	1	3.113	0.044
${\left(2,\;0,\;0,\;0,\;0\right)}_{2}$	1	1.968	0.459	${\left(2,\;2,\;0,\;2,\;0\right)}_{2}$	1	3.020	0.045	${\left(0,\;0,\;0,\;0,\;2\right)}_{2}$	5	1.599	0.570
${\left(3,\;0,\;0,\;0,\;0\right)}_{1}$	1	2.482	0.287	${\left(1,\;3,\;0,\;2,\;0\right)}_{2}$	1	3.016	0.048	${\left(2,\;2,\;0,\;0,\;2\right)}_{1}$	1	2.996	0.061
${\left(0,\;1,\;0,\;0,\;0\right)}_{2}$	37	1.106	0.710	${\left(0,\;1,\;1,\;2,\;0\right)}_{2}$	1	2.670	0.216	${\left(0,\;3,\;0,\;0,\;2\right)}_{2}$	2	2.852	0.138
${\left(1,\;1,\;0,\;0,\;0\right)}_{2}$	5	1.924	0.473	${\left(1,\;2,\;1,\;2,\;0\right)}_{1}$	1	3.006	0.055	${\left(0,\;1,\;1,\;0,\;2\right)}_{2}$	1	2.562	0.258
${\left(0,\;2,\;0,\;0,\;0\right)}_{2}$	7	1.878	0.487	${\left(1,\;3,\;2,\;2,\;0\right)}_{1}$	1	3.063	0.005	${\left(0,\;2,\;1,\;0,\;2\right)}_{1}$	1	2.824	0.151
${\left(1,\;2,\;0,\;0,\;0\right)}_{2}$	1	2.426	0.307	${\left(0,\;1,\;0,\;3,\;0\right)}_{2}$	1	2.704	0.202	${\left(3,\;4,\;2,\;0,\;2\right)}_{1}$	1	3.121	0.049
${\left(3,\;2,\;0,\;0,\;0\right)}_{1}$	1	2.945	0.091	${\left(0,\;1,\;1,\;3,\;0\right)}_{1}$	1	2.882	0.124	${\left(4,\;3,\;3,\;0,\;2\right)}_{1}$	1	3.205	0.093
${\left(0,\;3,\;0,\;0,\;0\right)}_{1}$	1	2.396	0.318	${\left(2,\;4,\;1,\;4,\;0\right)}_{1}$	1	3.149	0.065	${\left(0,\;1,\;0,\;1,\;2\right)}_{1}$	1	2.602	0.243
${\left(0,\;0,\;1,\;0,\;0\right)}_{2}$	7	0.962	0.750	${\left(0,\;0,\;0,\;0,\;1\right)}_{2}$	12	0.910	0.764	${\left(0,\;1,\;1,\;1,\;2\right)}_{1}$	2	2.818	0.153
${\left(1,\;0,\;1,\;0,\;0\right)}_{1}$	1	1.828	0.502	${\left(0,\;1,\;0,\;0,\;1\right)}_{2}$	3	1.744	0.527	${\left(2,\;2,\;1,\;1,\;2\right)}_{2}$	1	3.062	0.006
${\left(0,\;1,\;1,\;0,\;0\right)}_{1}$	6	1.779	0.517	${\left(1,\;1,\;0,\;0,\;1\right)}_{1}$	1	2.341	0.337	${\left(3,\;3,\;3,\;1,\;2\right)}_{1}$	1	3.193	0.088
${\left(1,\;1,\;1,\;0,\;0\right)}_{2}$	1	2.363	0.329	${\left(0,\;2,\;0,\;0,\;1\right)}_{1}$	1	2.308	0.348	${\left(0,\;0,\;0,\;2,\;2\right)}_{1}$	1	2.593	0.246
${\left(0,\;2,\;1,\;0,\;0\right)}_{2}$	2	2.332	0.340	${\left(1,\;2,\;0,\;0,\;1\right)}_{1}$	1	2.691	0.208	${\left(0,\;1,\;0,\;2,\;2\right)}_{2}$	1	2.842	0.143
${\left(1,\;0,\;2,\;0,\;0\right)}_{2}$	1	2.296	0.352	${\left(0,\;1,\;1,\;0,\;1\right)}_{1}$	1	2.237	0.372	${\left(0,\;2,\;0,\;2,\;2\right)}_{2}$	1	2.979	0.072
${\left(0,\;1,\;2,\;0,\;0\right)}_{2}$	5	2.262	0.364	${\left(0,\;1,\;2,\;0,\;1\right)}_{1}$	1	2.579	0.251	${\left(0,\;2,\;1,\;2,\;2\right)}_{1}$	1	3.035	0.033
${\left(0,\;2,\;2,\;0,\;0\right)}_{1}$	3	2.642	0.227	${\left(0,\;0,\;0,\;1,\;1\right)}_{1}$	1	1.725	0.533	${\left(0,\;3,\;3,\;3,\;2\right)}_{2}$	1	3.097	0.033
${\left(1,\;2,\;3,\;0,\;0\right)}_{1}$	1	2.986	0.068	${\left(1,\;1,\;0,\;1,\;1\right)}_{2}$	1	2.683	0.211	${\left(0,\;2,\;1,\;4,\;2\right)}_{1}$	1	3.066	0.003
${\left(0,\;1,\;4,\;0,\;0\right)}_{2}$	1	2.814	0.155	${\left(2,\;1,\;0,\;1,\;1\right)}_{2}$	1	2.905	0.112	${\left(1,\;4,\;1,\;4,\;2\right)}_{2}$	1	3.202	0.092
${\left(0,\;0,\;0,\;1,\;0\right)}_{1}$	11	1.080	0.717	${\left(2,\;2,\;3,\;1,\;1\right)}_{1}$	1	3.067	0.004	${\left(2,\;2,\;2,\;4,\;2\right)}_{1}$	1	3.193	0.088
${\left(0,\;1,\;0,\;1,\;0\right)}_{2}$	5	1.861	0.492	${\left(0,\;0,\;0,\;2,\;1\right)}_{1}$	1	2.284	0.356	${\left(0,\;0,\;0,\;0,\;3\right)}_{2}$	1	2.106	0.415
${\left(1,\;1,\;0,\;1,\;0\right)}_{1}$	1	2.415	0.311	${\left(1,\;1,\;0,\;2,\;1\right)}_{2}$	1	2.889	0.120	${\left(3,\;1,\;0,\;3,\;3\right)}_{2}$	1	3.080	0.018
${\left(0,\;2,\;0,\;1,\;0\right)}_{1}$	2	2.385	0.322	${\left(4,\;2,\;0,\;2,\;1\right)}_{2}$	1	3.071	0.009	${\left(1,\;0,\;0,\;4,\;3\right)}_{1}$	1	3.060	0.009
${\left(0,\;0,\;1,\;1,\;0\right)}_{1}$	1	1.761	0.522	${\left(0,\;0,\;1,\;2,\;1\right)}_{2}$	1	2.610	0.239	${\left(1,\;3,\;3,\;3,\;4\right)}_{2}$	1	3.434	0.186
${\left(0,\;1,\;1,\;1,\;0\right)}_{2}$	2	2.320	0.344	${\left(3,\;2,\;2,\;2,\;1\right)}_{2}$	1	3.083	0.021	${\left(2,\;4,\;4,\;3,\;4\right)}_{2}$	1	4.782	0.573
${\left(0,\;1,\;2,\;1,\;0\right)}_{2}$	1	2.634	0.230	${\left(0,\;0,\;0,\;3,\;1\right)}_{1}$	1	2.647	0.225	${\left(2,\;3,\;4,\;4,\;4\right)}_{1}$	1	4.762	0.568
${\left(0,\;0,\;0,\;2,\;0\right)}_{1}$	2	1.844	0.497	${\left(0,\;1,\;0,\;3,\;1\right)}_{1}$	2	2.873	0.128				
${\left(0,\;1,\;0,\;2,\;0\right)}_{2}$	1	2.374	0.326	${\left(1,\;4,\;3,\;3,\;1\right)}_{2}$	1	3.193	0.088				

**FIGURE 1:**
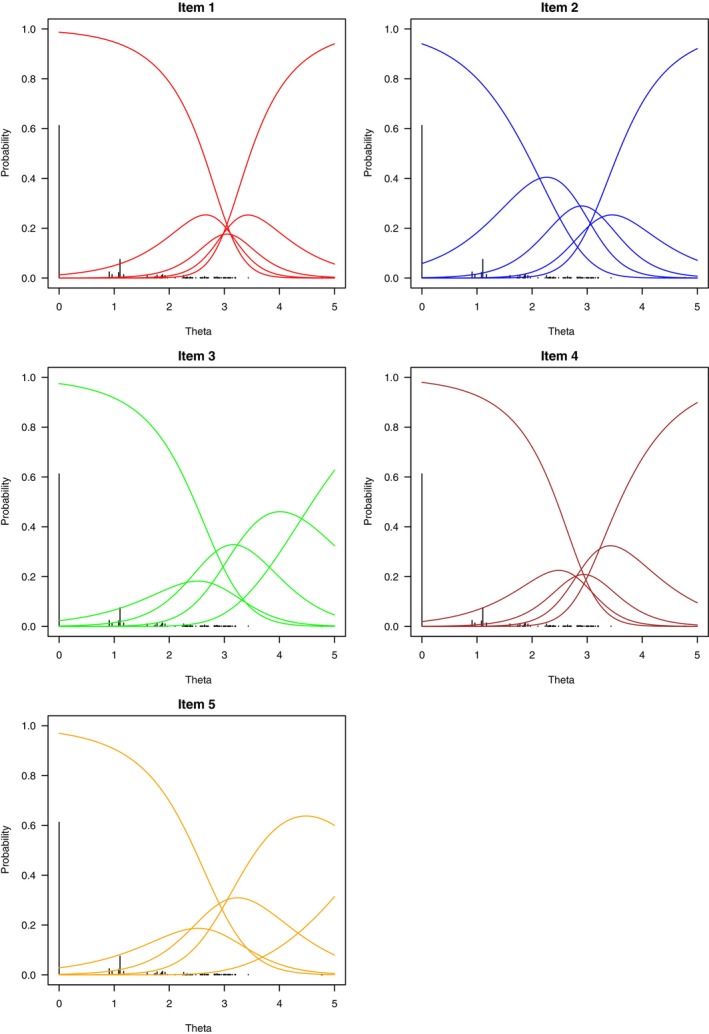
Estimated conditional probability distributions given θ for five‐item scores under the convexity‐constrained parameterization of the random effects generalized partial credit model fitted to the Dekovic (2003) data.

The number of observed score patterns is 85, and the number of theoretically possible score patterns is $5^{5}=3125$. The random effects generalized partial credit model is fitted to the data, and to include skewness and kurtosis parameters v is set to 6. The assumed subpopulation is defined by B=O. Since 0=(0,0,0,0,0)∈O, $y_{0}$ is conveniently set to 0. Since v=6, q=(v−2)/2=2. For convexity of the conditional cumulant‐generating function, the first polynomial is of degree q and the degree of the second polynomial is parsimoniously set to q−1. Thus, $p_{1}(\alpha^{\prime }\tilde{y})=\epsilon_{10}+\epsilon_{11}\alpha^{\prime }\tilde{y}+\epsilon_{12}{\left(\alpha^{\prime }\tilde{y}\right)}^{2}$, and $p_{2}(\alpha^{\prime }\tilde{y})=\epsilon_{20}+\epsilon_{21}\alpha^{\prime }\tilde{y}$. For identification, $\kappa_{1}=0$, $\epsilon_{10}=\frac{3}{5}$, and $\epsilon_{20}=\frac{4}{5}$. As a consequence, $\kappa_{2}=1$. In total, there are 28 parameters to be estimated, the $\sum_{i=1}^{5}m_{i}=5\cdot 4=20$ elements of β, the five elements of α, and $\epsilon_{11}$, $\epsilon_{12}$, and $\epsilon_{21}$. The parameter estimation results are given in Table 2. Note that the maximum likelihood estimate of $\delta_{is}$ is ${\hat{\delta }}_{is}=-{\hat{\beta }}_{is}/{\hat{\alpha }}_{i}$ if s=1 and ${\hat{\delta }}_{is}=\;\left({\hat{\beta }}_{i(s-1)}-{\hat{\beta }}_{is}\right)\;/{\hat{\alpha }}_{i}$ otherwise, where ${\hat{\beta }}_{is}$ and ${\hat{\alpha }}_{i}$ are the maximum likelihood estimates of $\beta_{is}$ and $\alpha_{i}$, for all i and s. The maximum likelihood estimate of $\omega_{is}$ is ${\hat{\omega }}_{is}=-{\hat{\beta }}_{is}/(s{\hat{\alpha }}_{i})$, for all i and s. In Figure 1, the conditional probability distributions of the five‐item scores given θ have been plotted.

**TABLE 2:** Maximum likelihood estimates of parameters of generalized partial credit model fitted to Dekovic (2003) data.

Parameter	Estimate	SE	Parameter	Estimate	SE	Parameter	Estimate	SE
$\beta_{11}$	−4.340	0.521	$\delta_{11}$	3.107	0.284	$\omega_{11}$	3.107	0.284
$\beta_{12}$	−8.705	1.440	$\delta_{12}$	3.125	0.369	$\omega_{12}$	3.116	0.210
$\beta_{13}$	−12.851	2.246	$\delta_{13}$	2.968	0.488	$\omega_{13}$	3.067	0.182
$\beta_{14}$	−17.020	3.142	$\delta_{14}$	2.984	0.651	$\omega_{14}$	3.046	0.192
$\beta_{21}$	−2.781	0.343	$\delta_{21}$	2.136	0.165	$\omega_{21}$	2.136	0.165
$\beta_{22}$	−6.514	1.026	$\delta_{22}$	2.868	0.188	$\omega_{22}$	2.502	0.106
$\beta_{23}$	−10.768	1.777	$\delta_{23}$	3.267	0.359	$\omega_{23}$	2.757	0.135
$\beta_{24}$	−14.717	2.689	$\delta_{24}$	3.033	0.496	$\omega_{24}$	2.826	0.131
$\beta_{31}$	−3.738	0.416	$\delta_{31}$	3.383	0.374	$\omega_{31}$	3.383	0.374
$\beta_{32}$	−6.293	1.147	$\delta_{32}$	2.312	0.384	$\omega_{32}$	2.847	0.177
$\beta_{33}$	−9.868	2.002	$\delta_{33}$	3.236	0.604	$\omega_{33}$	2.977	0.220
$\beta_{34}$	−14.733	3.210	$\delta_{34}$	4.403	1.601	$\omega_{34}$	3.333	0.386
$\beta_{41}$	−3.905	0.396	$\delta_{41}$	3.083	0.302	$\omega_{41}$	3.083	0.302
$\beta_{42}$	−7.433	1.079	$\delta_{42}$	2.785	0.283	$\omega_{42}$	2.934	0.168
$\beta_{43}$	−10.997	1.774	$\delta_{43}$	2.813	0.416	$\omega_{43}$	2.894	0.162
$\beta_{44}$	−15.083	2.589	$\delta_{44}$	3.226	0.528	$\omega_{44}$	2.977	0.162
$\beta_{51}$	−3.544	0.315	$\delta_{51}$	3.418	0.362	$\omega_{51}$	3.418	0.362
$\beta_{52}$	−6.030	0.818	$\delta_{52}$	2.397	0.330	$\omega_{52}$	2.907	0.187
$\beta_{53}$	−9.198	1.385	$\delta_{53}$	3.055	0.704	$\omega_{53}$	2.957	0.241
$\beta_{54}$	−15.030	2.833	$\delta_{54}$	5.625	1.870	$\omega_{54}$	3.624	0.451
$\alpha_{1}$	1.397	0.259	$\kappa_{3}$	−0.251	0.020			
$\alpha_{2}$	1.302	0.228	$\kappa_{4}$	0.046	0.011			
$\alpha_{3}$	1.105	0.215	$\kappa_{5}$	−0.006	0.003			
$\alpha_{4}$	1.267	0.217	$\lambda_{6}$	0.001	0.000			
$\alpha_{5}$	1.037	0.169						
$\epsilon_{11}$	−0.110	0.019						
$\epsilon_{12}$	0.005	0.002						
$\epsilon_{21}$	−0.074	0.005						

The maximum likelihood estimates of $\kappa_{3}$, $\kappa_{4}$, $\kappa_{5}$, and $\lambda_{6}$ are

$$\begin{array}{ll} {\hat{\kappa }}_{3} & =h_{1}({\hat{\epsilon }}_{1},{\hat{\epsilon }}_{2})=2{\hat{\epsilon }}_{10}{\hat{\epsilon }}_{11}+2{\hat{\epsilon }}_{20}{\hat{\epsilon }}_{21}=2\cdot \frac{3}{5}\cdot {\hat{\epsilon }}_{11}+2\cdot \frac{4}{5}\cdot {\hat{\epsilon }}_{21},\;\\ {\hat{\kappa }}_{4} & =2h_{2}({\hat{\epsilon }}_{1},{\hat{\epsilon }}_{2})=2({\hat{\epsilon }}_{11}^{2}+{\hat{\epsilon }}_{21}^{2}+2{\hat{\epsilon }}_{10}{\hat{\epsilon }}_{12})=2({\hat{\epsilon }}_{11}^{2}+{\hat{\epsilon }}_{21}^{2}+2\cdot \frac{3}{5}\cdot {\hat{\epsilon }}_{12}),\;\\ {\hat{\kappa }}_{5} & =6h_{3}({\hat{\epsilon }}_{1},{\hat{\epsilon }}_{2})=6\cdot 2{\hat{\epsilon }}_{11}{\hat{\epsilon }}_{12},\;\\ {\hat{\lambda }}_{6} & =24h_{4}({\hat{\epsilon }}_{1},{\hat{\epsilon }}_{2})=24\cdot {\hat{\epsilon }}_{12}^{2},\; \end{array}$$

respectively. The estimated standard errors of ${\hat{\delta }}_{is}$ and ${\hat{\omega }}_{is}$, for all i and s, ${\hat{\kappa }}_{3}$, ${\hat{\kappa }}_{4}$, ${\hat{\kappa }}_{5}$, and ${\hat{\lambda }}_{6}$ were obtained by the delta method.

**FIGURE 2:**
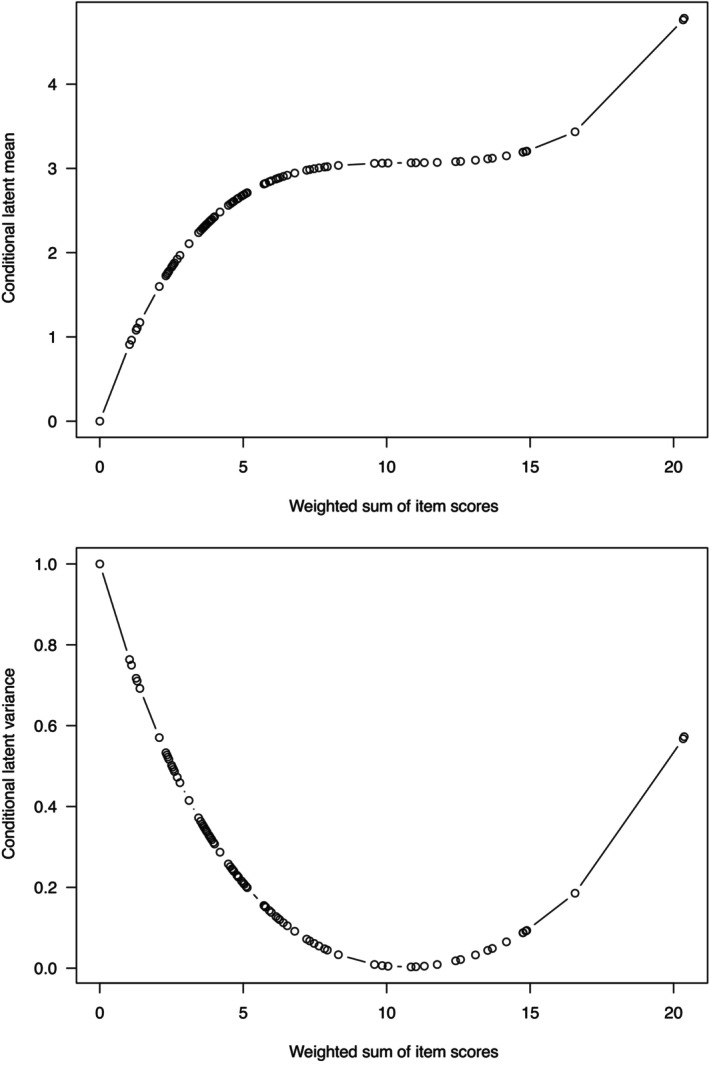
Estimated conditional mean (EAP) and variance of Θ given ${\hat{\alpha }}^{\prime }y$ under convexity‐constrained parameterization of random effects generalized partial credit model fitted to Dekovic (2003) data.

To test the goodness of fit of the random effects generalized partial credit model, the following likelihood ratio test is used (Hessen, 2023). First, score patterns are randomly assigned to $O_{1}$ and $O_{2}$, such that $O_{1}\cup O_{2}=O$ and $O_{1}\cap O_{2}=\emptyset$. Score patterns in Table 1 that were assigned to $O_{1}$ have subscript 1 and those assigned to $O_{2}$ have subscript 2. Let $\hat{l}$ be the overall maximum of the log‐likelihood function based on all y∈O, ${\hat{l}}_{1}$ the maximum of the log‐likelihood function based on all $y\in O_{1}$, ${\hat{l}}_{2}$ the maximum of the log‐likelihood function based on all $y\in O_{2}$, $n_{1}$ the sum of the observed frequencies of score patterns in $O_{1}$, and $n_{2}$ the sum of the observed frequencies of score patterns in $O_{2}$. Then, under the random effects generalized partial credit model, the value of

$$LR=2\left\{{\hat{l}}_{1}+n_{1}\ln\left(\frac{n_{1}}{n}\right)+{\hat{l}}_{2}+n_{2}\ln\left(\frac{n_{2}}{n}\right)-\hat{l}\right\}$$

is the sample value of a random variable having an asymptotic chi‐squared distribution with its degrees of freedom equal to the total number of free parameters of the random effects generalized partial credit model plus one (Hessen, 2023). For the present data and partition, $\hat{l}=-1055.679$, ${\hat{l}}_{1}=-371.522$, ${\hat{l}}_{2}=-392.198$, $n_{1}=363$, and $n_{2}=130$, which yields LR=15.110 on 29 degrees of freedom. The corresponding p‐value is .984. Thus, based on this likelihood ratio test, the random effects generalized partial credit model cannot be rejected. Next, the EAP estimates and estimated conditional variances of Θ have been calculated and are also given in Table 1. In the plots of Figure 1, the vertical black lines represent the relative frequencies of the EAP estimates and indicate positive skewness. In Figure 2, $K_{\Theta |y_{0}}^{\left(1\right)}({\hat{\alpha }}^{\prime }\tilde{y})={\hat{\mu }}_{y}$ and $K_{\Theta |y_{0}}^{\left(2\right)}({\hat{\alpha }}^{\prime }\tilde{y})={\hat{\sigma }}_{y}^{2}$ were plotted against ${\hat{\alpha }}^{\prime }y$, for all y. Obviously, $K_{\Theta |y_{0}}^{\left(1\right)}({\hat{\alpha }}^{\prime }\tilde{y})$ is strictly increasing, and $K_{\Theta |y_{0}}^{\left(2\right)}({\hat{\alpha }}^{\prime }\tilde{y})$ is non‐negative.

The maximum likelihood estimates of the first four non‐central moments of Θ in the population defined by O are ${\hat{\mu }}_{1O}^{\prime }=.749$, ${\hat{\mu }}_{2O}^{\prime }=2.472$, ${\hat{\mu }}_{3O}^{\prime }=5.035$, and ${\hat{\mu }}_{4O}^{\prime }=17.784$. Consequently, the maximum likelihood estimates of the first four cumulants of Θ in the population defined by O are ${\hat{\kappa }}_{1O}=.749$, ${\hat{\kappa }}_{2O}=1.911$, ${\hat{\kappa }}_{3O}=0.322$, and ${\hat{\kappa }}_{4O}=-27.848$. Thus, the maximum likelihood estimates of the mean and variance of Θ in the population defined by O are .749 and 1.911, and the distribution of Θ in the population defined by O seems positively skewed and platykurtic.

## 8 DISCUSSION

The random effects generalized partial credit model can be fitted to data with or without the specification of a latent variable distribution in the population. If the latent variable distribution is completely specified, then the model has $\sum_{i=1}^{k}m_{i}+k$ free parameters. If the latent variable distribution is not specified, then the model has v−2 more free parameters, where v≥2 and might be larger than $\sum_{i=1}^{k}m_{i}$, which is the theoretical maximum of v under the random effects partial credit model if all theoretically possible values of total score $\tilde{y}$ are observable. Since the total number of free parameters cannot exceed the number of free parameters of the saturated model, which is the cardinality of B minus one, v cannot exceed the cardinality of B plus one minus $\sum_{i=1}^{k}m_{i}+k$ but might be less due to a smaller cardinality of O. In practice, however, it is not necessary to know the maximum value of v. Ideally, the specified value of v is as small as possible and yields a good fitting model that replicates well. It is therefore recommended to try out different values of v and determine the most parsimonious value of v by goodness‐of‐fit tests, chi‐squared difference tests, and especially cross validation (Hessen, 2023).

The procedure proposed to find the maximum likelihood estimates of the parameters of the random effects generalized partial credit model is the numerical BFGS procedure. In each iteration, the BFGS algorithm uses an updated approximated Hessian. The final approximated Hessian is subsequently used to calculate the estimated standard errors of all estimates of the free parameters and functions thereof using the delta method. It is well known that the required calculations for these estimated standard errors are prone to numerical errors. Thus, the reported estimates of the standard errors should be handled with caution. More precise estimates of the standard errors would be obtained if the analytical Hessian were available and used. Unfortunately, however, the derivation and implementation of the analytical Hessian are difficult and have not been done yet.

The expressions and procedures proposed in this paper are a basis for several extensions. The convexity‐constrained parameterization of the random effects generalized partial credit model can be extended to a multigroup model, which can be used in test equating, in assessing measurement invariance, in studying latent variable differences, and in testing for differential item functioning. The advantage of such a multigroup model for these applications would be the possibility to independently estimate properties of the distributions of the latent variable in several (sub)populations. Furthermore, the convexity‐constrained model can be extended to a multidimensional model, which can be used for dimension reduction or factor analysis of polytomously scored items. Advantages of such a multidimensional extension over existing multidimensional latent variable models would be the possibility to fit a multidimensional latent variable model without assuming a specific form for the multivariate distribution of the latent variables and the possibility to take into account and estimate higher‐order associations between the latent variables than just the usual two‐way associations. In addition, the convexity‐constrained model can be extended to a longitudinal model, which can be used to analyse repeatedly administered polytomously scored items and study measurement invariance or latent change across time points. An advantage of such a longitudinal extension would be the possibility to fit a latent variable distribution‐free longitudinal model for polytomously scored items that assumes conditional independence between the item scores at each time point but takes into account possible auto‐associations of the item scores between time points.

Although all these extensions seem straightforward, the development of each extension will probably have its own specific challenges. The multigroup extension seems the least challenging. The only challenge for this extension seems to be the writing of software code that gives the flexibility to fit a multigroup model with all sorts of parameter constraints across groups. The development of a multidimensional extension seems more theoretically challenging because in the case of a conditional multivariate distribution of latent variables, the Hessian of the conditional multivariate cumulant‐generating function must be constrained to be positive semi‐definite. Finally, two challenges for the longitudinal extension seem to be the theoretical incorporation of auto‐associations of item scores between time points and the writing of software code that gives the flexibility to fit a longitudinal model with all sorts of parameter constraints across time points.

## AUTHOR CONTRIBUTIONS

**David J. Hessen:** conceptualization; software; methodology; writing – review and editing.

## CONFLICT OF INTEREST STATEMENT

The author declares that there is no conflict of interest.

## Data Availability

The data that support the findings are available in Table 1 of the manuscript and from the repository RE‐GPCM at https://github.com/djhessen.

## Appendix

The log‐likelihood function for the model in Eq. 32 is given by

$$\ln L=\beta^{\prime }\tilde{n}+\underset{y}{\sum }n_{y}K_{\Theta |y_{0}}(\alpha^{\prime }\tilde{y})-n\ln\left\{\underset{y}{\sum }b(y)\right\},$$

where $b(y)=\exp\;\left\{\beta^{\prime }\tilde{x}+K_{\Theta |y_{0}}(\alpha^{\prime }\tilde{y})\right\}$. The derivatives of lnL with respect to β, α, and $\epsilon_{uj}$ are given by

$$\begin{array}{ll} \frac{\partial \ln L}{\partial \beta } & =\tilde{n}-\frac{n}{\sum_{y}b(y)}\underset{y}{\sum }b(y)\tilde{x},\;\\ \frac{\partial \ln L}{\partial \alpha } & =\underset{y}{\sum }n_{y}\frac{\partial }{\partial \alpha }\{K_{\Theta |y_{0}}(\alpha^{\prime }\tilde{y})\}-\frac{n}{\sum_{y}b(y)}\underset{y}{\sum }b(y)\frac{\partial }{\partial \alpha }\{K_{\Theta |y_{0}}(\alpha^{\prime }\tilde{y})\},\;\\ \frac{\partial \ln L}{\partial \epsilon_{uj}} & =\underset{y}{\sum }n_{y}\frac{\partial }{\partial \epsilon_{uj}}\{K_{\Theta |y_{0}}(\alpha^{\prime }\tilde{y})\}-\frac{n}{\sum_{y}b(y)}\underset{y}{\sum }b(y)\frac{\partial }{\partial \epsilon_{uj}}\{K_{\Theta |y_{0}}(\alpha^{\prime }\tilde{y})\}.\; \end{array}$$

The derivatives of $K_{\Theta |y_{0}}(\alpha^{\prime }\tilde{y})$ with respect to α and $\epsilon_{uj}$ are

$$\begin{array}{ll} \frac{\partial }{\partial \alpha }\{K_{\Theta |y_{0}}(\alpha^{\prime }\tilde{y})\} & =\lambda_{1}\tilde{y}+\overset{2q}{\underset{c=0}{\sum }}h_{c}(\epsilon_{1},\epsilon_{2})\frac{1}{c+1}{\left(\alpha^{\prime }\tilde{y}\right)}^{c+1}\tilde{y},\;\\ \frac{\partial }{\partial \epsilon_{uj}}\{K_{\Theta |y_{0}}(\alpha^{\prime }\tilde{y})\} & =\overset{2q}{\underset{c=0}{\sum }}\frac{\partial }{\partial \epsilon_{uj}}\{h_{c}(\epsilon_{1},\epsilon_{2})\}\frac{1}{c+1}\frac{1}{c+2}{\left(\alpha^{\prime }\tilde{y}\right)}^{c+2},\; \end{array}$$

where $\frac{\partial }{\partial \epsilon_{uj}}\{h_{c}(\epsilon_{1},\epsilon_{2})\}=2\epsilon_{u(c-j)}$, for j≤c≤j+q, and $\frac{\partial }{\partial \epsilon_{uj}}\{h_{c}(\epsilon_{1},\epsilon_{2})\}=0$ otherwise.

The first four conditional non‐central moments of Θ as polynomial functions of the first four conditional cumulants of Θ given Y=y are

$$\begin{array}{ll} \mu_{1y}^{\prime } & =\kappa_{1y},\;\\ \mu_{2y}^{\prime } & =\kappa_{2y}+\kappa_{1y}^{2},\;\\ \mu_{3y}^{\prime } & =\kappa_{3y}+3\kappa_{2y}\kappa_{1y}+\kappa_{1y}^{3},\;\\ \mu_{4y}^{\prime } & =\kappa_{4y}+4\kappa_{3y}\kappa_{1y}+3\kappa_{2y}^{2}+6\kappa_{2y}\kappa_{1y}^{2}+\kappa_{1y}^{4},\; \end{array}$$

for all y.
